# Relationship between Phase Occurrence, Chemical Composition, and Corrosion Behavior of as-Solidified Al–Pd–Co Alloys

**DOI:** 10.3390/ma12101661

**Published:** 2019-05-22

**Authors:** Marián Palcut, Libor Ďuriška, Ivona Černičková, Sandra Brunovská, Žaneta Gerhátová, Martin Sahul, Ľubomír Čaplovič, Jozef Janovec

**Affiliations:** Faculty of Materials Science and Technology in Trnava, Slovak University of Technology in Bratislava, J. Bottu 25, 917 24 Trnava, Slovakia; marian.palcut@stuba.sk (M.P.); libor.duriska@stuba.sk (L.Ď.); ivona.cernickova@stuba.sk (I.Č.); brunovskas@gmail.com (S.B.); zaneta.gerhatova@stuba.sk (Ž.G.); marian.sahul@stuba.sk (M.S.); lubomir.caplovic@stuba.sk (Ľ.Č.)

**Keywords:** aluminum alloys, phase characterization, electrochemical corrosion, de-alloying

## Abstract

The microstructure, phase constitution, and corrosion performance of as-solidified Al_70_Pd_25_Co_5_ and Al_74_Pd_12_Co_14_ alloys (element concentrations in at.%) have been investigated in the present work. The alloys were prepared by arc-melting of Al, Pd, and Co lumps in argon. The Al_74_Pd_12_Co_14_ alloy was composed of structurally complex ε_n_ phase, while the Al_70_Pd_25_Co_5_ alloy was composed of ε_n_ and δ phases. The corrosion performance was studied by open circuit potential measurements and potentiodynamic polarization in aqueous NaCl solution (3.5 wt.%). Marked open circuit potential oscillations of the Al_70_Pd_25_Co_5_ alloy have been observed, indicating individual breakdown and re-passivation events on the sample surface. A preferential corrosion attack of ε_n_ was found, while the binary δ phase (Al_3_Pd_2_) remained free of corrosion. A de-alloying of Al from ε_n_ and formation of intermittent interpenetrating channel networks occurred in both alloys. The corrosion behavior of ε_n_ is discussed in terms of its chemical composition and crystal structure. The corrosion activity of ε_n_ could be further exploited in preparation of porous Pd–Co networks with possible catalytic activity.

## 1. Introduction

Alloys with nominal chemical composition of approximately Al-30 at.% TM (TM stands for one or more transition metals) constitute a specific group of materials called complex metallic alloys (CMAs). These metallic materials contain, besides classical crystalline phases with simple unit cells, structurally complex intermetallic phases (SCIPs) [1]. The SCIPs are composed of giant unit cells and lack translational symmetry. Because of their complex atomic structure, the SCIPs are appealing for thin film applications, coatings, and reinforcement phases in composites [2].

**Figure 1:**
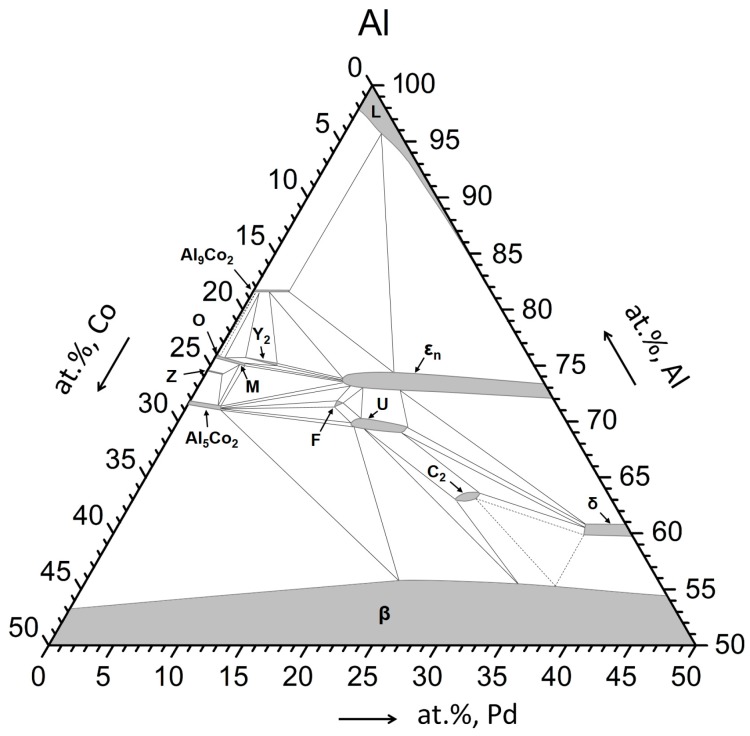
Isothermal section of the Al–Pd–Co phase diagram at 790 °C, redrawn from Reference [3].

The phase equilibria in the Al–Pd–Co system have been studied by Yurechko et al. [3,4], Černičková et al. [5,6], and Ďuriška et al. [7]. The authors observed six stable ternary phases (W, Y_2_, U, V, F, C_2_) and a structurally complex ε-family. Selected phases occurring in the Al–Pd–Co system are summarized in Table 1 [3,8]. Their homogeneity ranges at 790 °C are shown in the corresponding isothermal phase diagram section (Figure 1). The cluster-based orthorhombic decagonal quasicrystalline approximant of the ε-family consists of five structures: two binary (ε_6_, ε_28_) and three ternary (ε_16_, ε_22_, ε_34_). Since the ε-family is considered to be a single phase from the thermodynamic point of view, it has been briefly denoted as ε_n_. Although two lattice parameters (*a* and *b*) are identical for each structure within the family, the third lattice parameter (*c*) differs for each of the structures since it is associated with the cluster arrangement [3,9]. Contrary to Al–Pd alloys [10], Co was observed to substitute Pd in ε_n_ in ternary Al–Pd–Co alloys. The Co solubility in ε_n_ is up to approximately 15 at.% at 790 °C [3]. The Al–Pd–TM ε_n_ phase is predominantly diamagnetic and has a good electrical conductivity. This phase is brittle and can be easily powdered. Furthermore, it contains Pd, a catalytically active element, which, in combination with a unique crystal structure, provides a variety of different adsorption sites. As such, the Al–Pd–Co SCIPs are interesting for catalytic applications [11].

The corrosion activity of Al-based SCIPs is relatively unknown. It has been found that the electrochemical properties of CMAs differ from those of aluminum metal [12]. The previous studies of Al–Cu–Fe [13,14], Al–Cr–Fe [15], and Al–Cu–Fe–Cr [16] CMAs indicated that the relative amount of alloy phases and their chemical compositions had a major influence on their electrochemical behavior. It was presented that Cr additions significantly improved the corrosion resistance of Al–Cr–Fe and Al–Cu–Fe–Cr alloys [16]. Recent studies of Al–Co CMAs [17,18,19,20,21] have shown that the relative amounts of the alloy’s phases and electrical contact between them played an important role in their corrosion performance. The anodic dissolution of different alloy phases was found to take place by a galvanic mechanism. The electrochemical nobility of Al–Co SCIPs was found to increase with increasing Co concentration. The phase crystal structure had only a secondary influence. An exception, however, was found for the structurally complex Z-Al_3_Co phase. This phase was found to be more corrosion resistant compared to Al_5_Co_2_ in chloride-containing environments [19]. The reason for this behavior could stem from the complex crystal structure of Z-Al_3_Co, formed by a complex monoclinic unit cell containing large pentagons composed of six small pentagons of monoclinic Al_13_Co_4_. The complex structure of this phase is probably stabilized by vacancies. The vacancies may influence the Co diffusivity leading to a protective layer formation on the sample surface.

The corrosion behavior of Al–Pd alloys in various solutions has been studied in References [22,23,24,25]. The results showed a preferential Al dissolution from ε_n_ (~Al_3_Pd). The corrosion attack of the structurally complex ε_n_ in the Al–Pd alloys led to the formation of a porous, channel-like network [22,23,24,25]. This phenomenon is known as electrochemical de-alloying [26], i.e., a corrosion-driven process during which an alloy is decomposed by selective dissolution of the most electrochemically active element (Al). This process results in the formation of nano-porous metal networks composed of noble elements. In the NaCl aqueous solution, the de-alloying of Al–Pd alloys was found to be more pronounced in as-solidified alloys compared to as-annealed samples [24]. The de-alloying of Al–TM alloys has attracted much attention in recent years as a versatile tool for creating nano-porous metal networks with high catalytic activity [25]. Nano-porous ribbons of Pd, Au, Pt, and other precious metals have been fabricated through chemical de-alloying of rapidly solidified Al-based alloys under free corrosion conditions [27].

In the present work, the corrosion performance of Al_70_Pd_25_Co_5_ and Al_74_Pd_12_Co_14_ alloys (element concentrations are given in at.%) have been studied by potentiodynamic polarization in 3.5 wt.% NaCl aqueous solution for the first time. The aim of this work is to investigate the effect of both phase occurrence and chemical composition on the alloy’s corrosion behavior. Furthermore, the effect of Co concentration on the corrosion behavior of ε_n_ is studied.

## 2. Materials and Methods

The alloys with nominal compositions Al_70_Pd_25_Co_5_ and Al_74_Pd_12_Co_14_ were prepared by repeated arc-melting of Al, Pd, and Co granules (purity of 99.95%) in argon. After melting, the alloys were rapidly solidified on a water-cooled copper mold, cast in epoxy resin, and metallographically prepared by wet grinding and polishing down to a surface roughness of 1 μm. The as-solidified alloy’s phase constitution and microstructure were studied by room temperature X-ray diffraction (XRD) and scanning electron microscopy (SEM), respectively. During XRD experiments, a Panalytical Empyrean PIXCel 3D diffractometer (Malvern Panalytical Ltd., Malvern, UK) with Bragg–Brentano geometry and Co Kα1,2 radiation was used. The measurements were conducted in the 2θ range between 20° and 60°, with the step size 0.0131° and the counting time 98 s per step. For the microstructure observation, a JEOL JSM-7600F scanning electron microscope (JEOL, Akishima, Tokyo, Japan), equipped with an Oxford Instruments X-max 50 spectrometer (Oxford Instruments, Abingdon, UK) and operated by the INCA software (version 5.04), was employed. The microscope was operated at the acceleration voltage of 20 kV. The scanning was performed in regimes of secondary (SEI) and backscattered (BEI) electrons. Furthermore, a scanning transmission electron microscope JEOL JEM ARM200F (JEOL, Akishima, Tokyo, Japan), operated at 200 kV and equipped with a high-angle annular dark-field detector (HAADF/STEM), was employed to obtain HAADF images. The two-dimensional (2D) projections of crystal structures were calculated in PowderCell software (version 2.4) using the data derived from References [8,28,29].

The corrosion experiments were conducted at room temperature (21 ± 2 °C) in a 500 ml glass vessel filled with an aqueous electrolyte. A three-electrode setup was used. The working electrode consisted of the polished surface of the Al–Pd–Co alloy with an exposed area of about 1 cm^2^. A silver–silver chloride electrode immersed in a saturated sodium chloride solution (saturated Ag/AgCl electrode) was used as a reference electrode. The counter electrode was a platinum mesh (2 × 2 cm^2^). The corrosion experiments were conducted in an aqueous NaCl solution (concentration 0.6 mol dm^−3^). The solution was prepared immediately before the experiment by dissolving 35 g of NaCl in 1 liter of de-ionized water (conductivity <20 μS). The electrolyte was not de-aerated before the experiment to simulate real environmental conditions. The progress of the reaction was controlled by a PGU 10 V-1A-IMP-S potentiostat/galvanostat from Jaissle Electronic Ltd. (Waiblingen, Germany).

The surface topography of the corroded samples was analyzed by a Zeiss LSM 700 confocal laser scanning microscope (CLSM, Zeiss, Oberkochen, Germany). The ZEN 2009 software was used for the three-dimensional topographical resolution.

## 3. Results and Discussion

### 3.1. Microstructure and Phase Occurrence before Corrosion Testing

**Figure 2:**
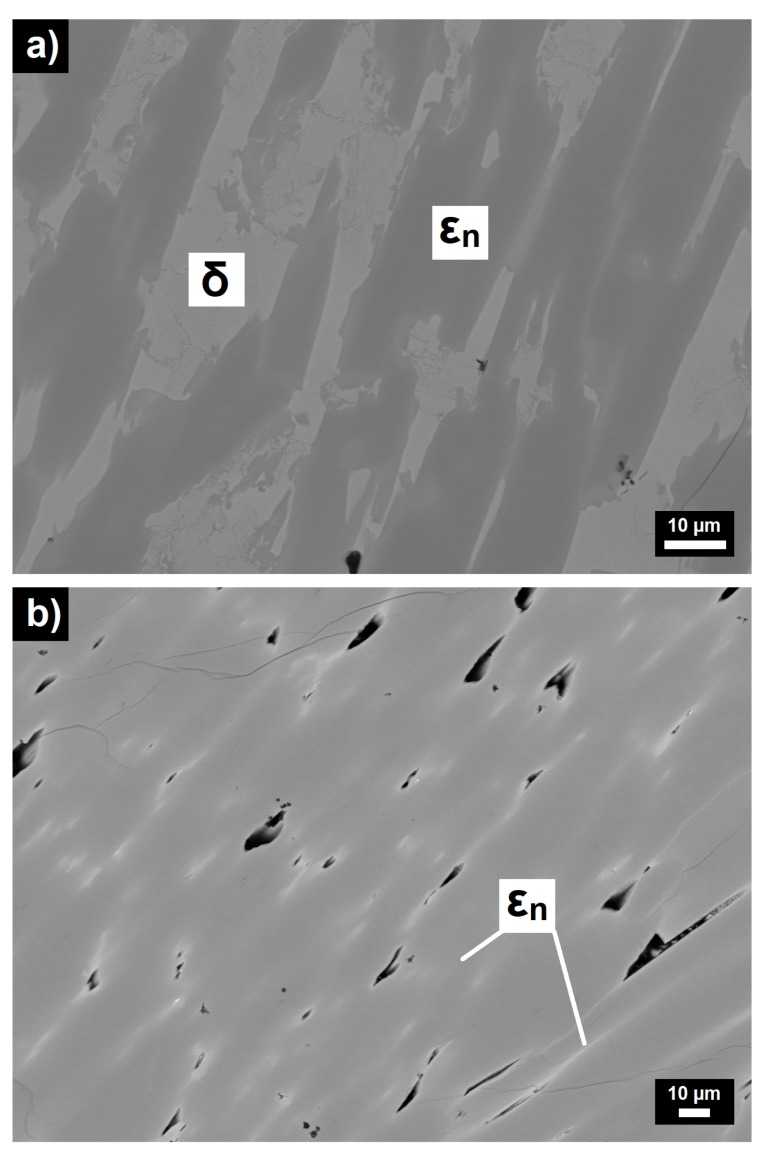
BEI/SEM images of microstructure constituents in as-solidified Al_70_Pd_25_Co_5_ (**a**) and Al_74_Pd_12_Co_14_ (**b**) alloys. Black areas in (**b**) correspond to pores. Phases assigned to particular constituents are also marked.

**Figure 3:**
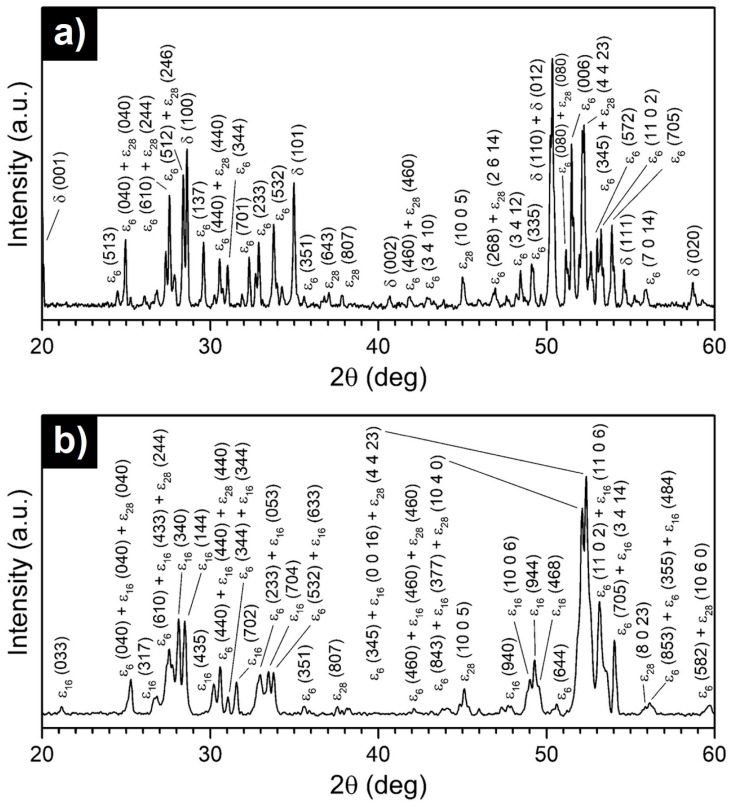
XRD diffraction patterns of as-solidified Al_70_Pd_25_Co_5_ (**a**) and Al_74_Pd_12_Co_14_ (**b**) alloys.

The microstructures of the as-solidified Al_70_Pd_25_Co_5_ and Al_74_Pd_12_Co_14_ alloys are illustrated in Figure 2. The XRD patterns corresponding to the above alloys are given in Figure 3a,b, respectively. The metal concentrations of microstructure constituents determined by SEM/EDX and their phase assignments are presented in Table 2.

The microstructure of the Al_70_Pd_25_Co_5_ alloy consisted of two different constituents (Figure 2a). The images were acquired in a BEI regime and therefore the bright regions have a higher Pd concentration compared to the dark constituents. The metal concentrations and volume fractions of the bright-grey microstructure constituent (Table 2) indicate that it corresponds to the δ phase (Al_3_Pd_2_). This assumption was also confirmed by X-ray diffraction (Figure 3a). The visually and chemically homogeneous dark-grey constituent was identified to be a mixture of ε_n_ structures (Figure 3a). To index diffraction peaks of particular ε_n_ structures, the data derived from References [8,28,29] were used.

**Figure 4:**
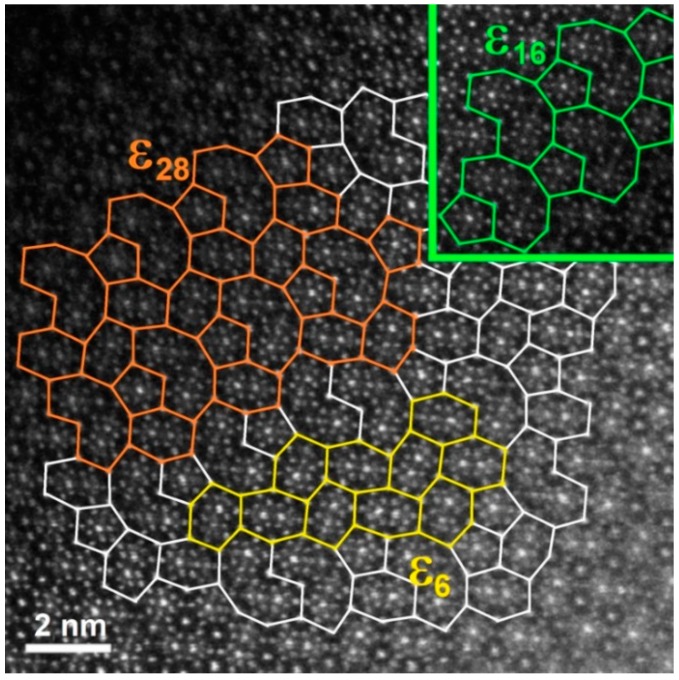
A high-angle annular dark-field (HAADF)/STEM image of the atomic structure of the as-solidified Al_74_Pd_12_Co_14_ alloy. Phason tiles, i.e., hexagon, pentagon, and banana-shaped nonagons, are highlighted by solid lines. Yellow, green, and orange structural motifs correspond to ε_6_, ε_16_, and ε_28_, respectively. For the color interpretation of this figure, the reader is referred to the web version of this article.

**Figure 5:**
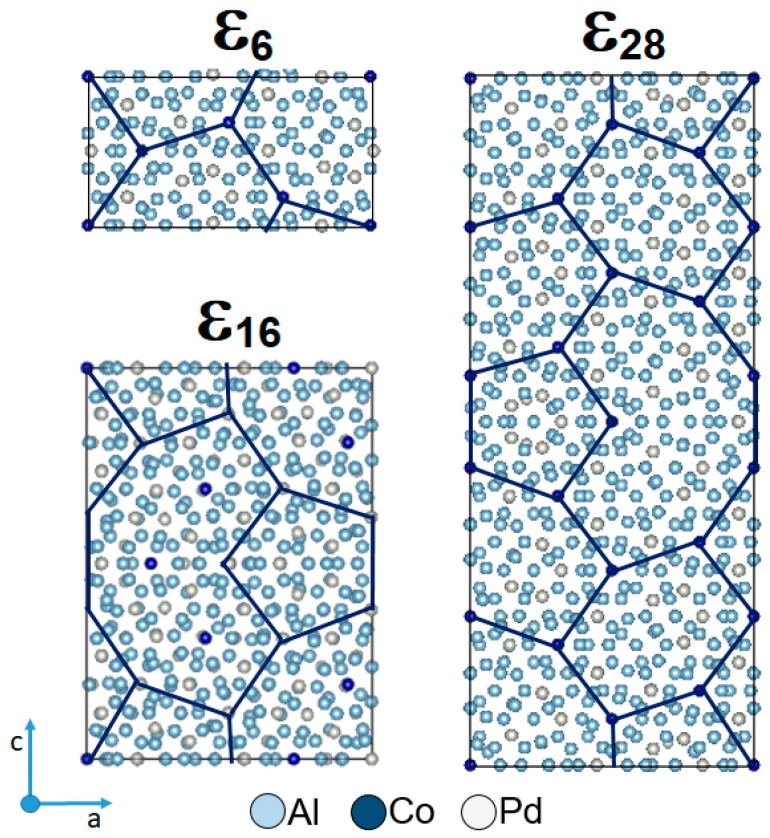
Two-dimensional projection of the crystal structure of ε_6_, ε_16_, and ε_28_. The phason tiling is denoted by dark-blue lines. For the color interpretation of this figure, the reader is referred to the web version of this article.

In the XRD pattern of the Al_74_Pd_12_Co_14_ alloy (Figure 3b), a combination of ε_6_, ε_16_, and ε_28_ structures was identified. In the related microstructure image, however, a chemically heterogeneous constituent has been observed (Figure 2b). The dark-grey areas had an increased Co concentration, while the bright areas showed a higher Pd concentration compared to the dark-grey areas. The atomic structure of the as-solidified Al_74_Pd_12_Co_14_ alloy was observed using HAADF/STEM. Three different structural motives have been recognized in the atomic structure of this alloy (ε_6_, ε_16_, and ε_28_, Figure 4). For each ε_n_ structure, specific combinations of phason tiles are characteristic. ε_6_ is formed by hexagons only, ε_16_ is represented by the combination of pentagons and nonagons, while ε_28_ comprises all three types of tiles. It has been suggested that transitions between various structures of the ε-family could be associated with a small rearrangement of clusters, resulting in changes in the occurrence and/or configuration of phason tiles. The arrangement of tiles in particular ε_n_ structures, observed experimentally in this work, was also calculated using the data derived from References [8,28,29]. The 2D projection of the ε_6_, ε_16_, and ε_28_ structures, presented in Figure 5, is in a good agreement with the HAADF/STEM image.

Structures of ε_6_ and ε_28_ were reported to be binary structural variants of ε_n_, while ε_16_ has been described as a ternary ε_n_ structure [3,10,29,30]. In the latter structure, Co atoms substitute Pd. Therefore, the dark-grey areas (Figure 2b, Table 2), enriched with Co from the Co–Pd balance point of view, could be assigned to the ternary ε_16_ structure in the as-solidified Al_74_Pd_12_Co_14_ alloy. Similarly, the bright areas in Figure 2b could be ascribed to the mixture of ε_6_ and ε_28_ structures, which lie closer to the Al–Pd binary edge of the Al–Pd–Co ternary system. The bright areas were located preferentially around pores. The pores were formed on the grain boundaries during solidification due to shrinking. Co and Pd concentrations of ε_n_ changed since de-mixing took place during solidification. The Pd concentration in ε_n_ increased towards the grain boundary. Thus, the Pd-rich ε_n_ (ε_6_ + ε_28_) were located preferentially around pores. The Co-rich ε_n_ (ε_16_) was located in the center of the grain as this phase structure solidified from the melt. The overall chemical composition of the ε_n_ phase in the Al_74_Pd_12_Co_14_ alloy is presented in Table 2. Due to the presence of ε_16_, the ε_n_ phase in the Al_74_Pd_12_Co_14_ alloy had a significantly higher Co concentration compared to the Al_70_Pd_25_Co_5_ alloy where the ternary ε_16_ phase has not been identified.

**Figure 6:**
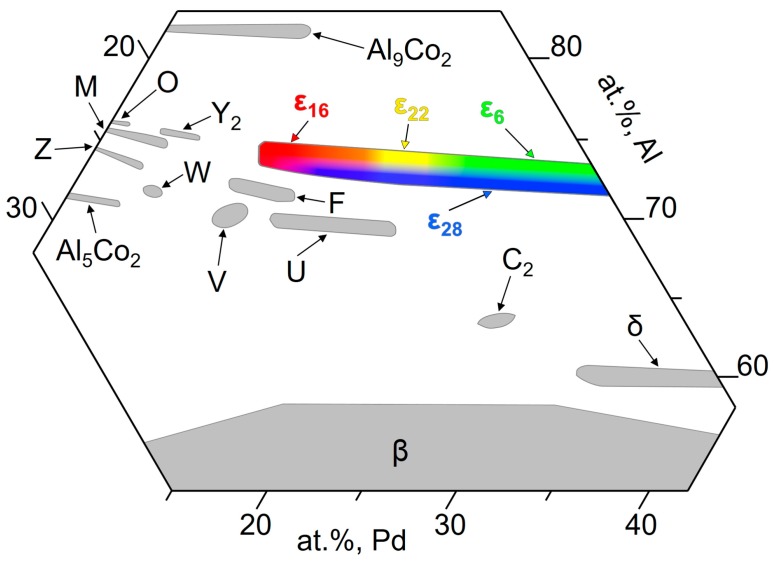
Schematic positions of binary and ternary phases in the isothermal section of a partial Al–Pd–Co diagram, redrawn from Reference [3]. For the color interpretation of this figure, the reader is referred to the web version of this article.

The distributions of particular structures within the ε_n_ phase were previously studied in the Al–Pd and Al–Pd–Co systems; however, the exact boundaries between structures have not been determined yet. In the Al–Pd system, Yurechko et al. [10] proposed a hypothetical double-phase area (ε_6_ + ε_28_) in between two single-phase areas (ε_6_ and ε_28_). In the partial phase diagram published by Grushko [31], ε_6_ and ε_28_ have been positioned in a common “single-phase (ε_6_ + ε_28_)” area consisting of two presumably separated subareas adherent to the particular structures. Earlier, the same distribution of ε_6_ and ε_28_ was studied by Balanetskyy et al. [28] in the Al–Pd–Fe system at 750 °C. Moreover, the homogeneity ranges of ε_16_ and ε_22_ have been defined. However, the strict boundaries between particular structures have not been described. In the Al–Pd–Co system, Yurechko et al. [3] estimated the boundaries of all the structures within the ε-family. Considering the results obtained using HAADF/STEM in this work and in [29], it can be assumed that the transitions between particular structures are rather open as schematically highlighted in gradient colors (green, red, yellow, and blue) in Figure 6. As follows from this figure, several ε_n_ structures in the transient area can coexist. This situation can also be seen in the microstructure of the Al_74_Pd_12_Co_14_ alloy. The dark-grey areas, corresponding to the ε_16_ structure, fluently transformed to the bright areas represented by the mixture of ε_6_ and ε_28_ structures (Figure 2b). The chemical composition of ε_6_ is very close to the composition of ε_28_. Consequently, this bright-grey microstructure constituent in the Al_70_Pd_25_Co_5_ alloy (Figure 2a) can be considered to be homogeneous. Individual ε_6_ and ε_28_ structures can be recognized in the HAADF/STEM image only (Figure 4).

### 3.2. Corrosion Behavior

**Figure 7:**
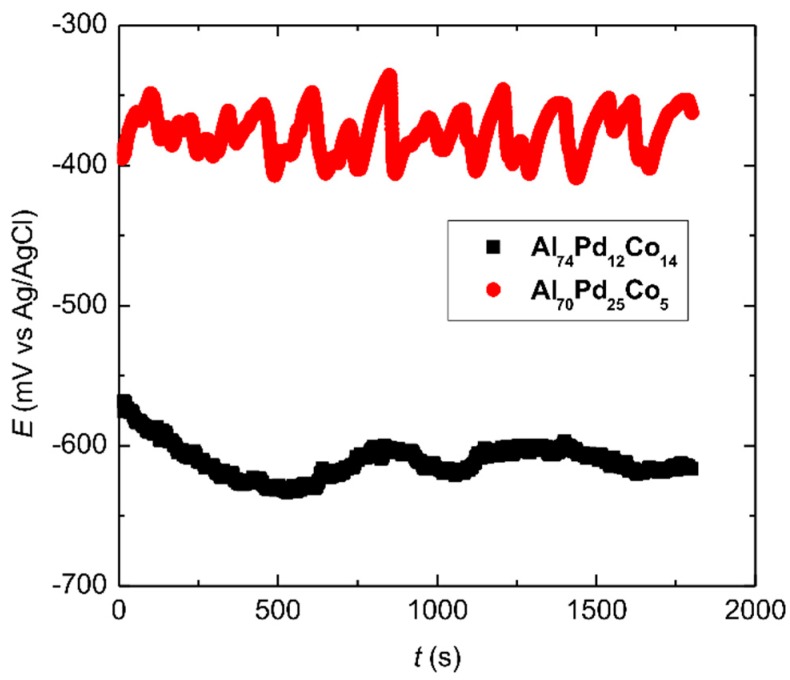
Open circuit potential of the as-solidified Al_74_Pd_12_Co_14_ and Al_70_Pd_25_Co_5_ alloys in 0.6 M NaCl. For the color interpretation of this figure, the reader is referred to the web version of this article.

Immediately after the sample’s immersion in aqueous NaCl, an open circuit potential (OCP) was recorded. The OCPs of the alloys are presented in Figure 7. A distinct behavior has been observed. While the OCP of the Al_74_Pd_12_Co_14_ alloy was relatively stable over time, irregular oscillations for the Al_70_Pd_25_Co_5_ alloy have been found. Furthermore, the OCPs of the Al_70_Pd_25_Co_5_ alloy were less negative and a difference of more than 200 mV was found compared to the Al_74_Pd_12_Co_14_ alloy. 

The as-solidified Al_74_Pd_12_Co_14_ alloy is a single-phase alloy. The OCP of this alloy therefore corresponds to the electrochemical activity of ε_n_. The Al_70_Pd_25_Co_5_ alloy, on the other hand, is a double-phase alloy composed of ε_n_ and δ (Al_3_Pd_2_). The less negative OCP of this alloy indicates a higher electrochemical potential of δ. Because of the potential difference between ε_n_ and δ, local galvanic cells may have been formed on the surface of the Al_70_Pd_25_Co_5_ alloy.

Every physical contact between δ and ε_n_ corresponds to an elementary galvanic corrosion cell. During galvanic corrosion, there is a net current flow between the cathodic microstructure constituent (δ) and its adjacent matrix (ε_n_). The metal ions dissolve into the solution on the anode and electrons released flow to the micro-cathodic area for the reduction process. This causes a redistribution of electrical charge between anodic (ε_n_) and cathodic areas (δ), thereby leading to a variation of the OCP. As the OCP is measured at the tip of the Haber–Luggin capillary, it represents the overall contributions of all elementary galvanic cells on the sample surface [32]. These contributions are not correlated. A high number of elementary galvanic cells between ε_n_ and δ co-exist with each other in the microstructure of the Al_70_Pd_25_Co_5_ alloy (Figure 2a). Their interactions are combined and contribute to the overall corrosion behavior of this alloy.

**Figure 8:**
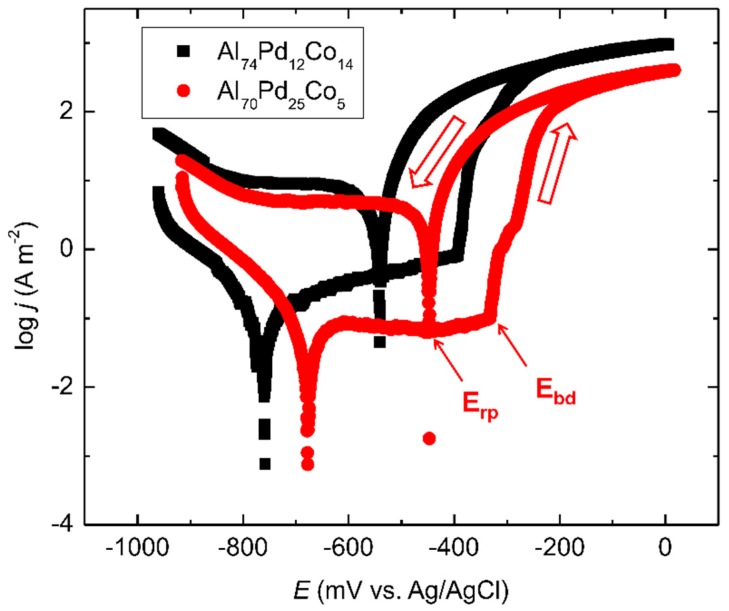
Cyclic potentiodynamic polarization curves of the as-solidified Al_74_Pd_12_Co_14_ and Al_70_Pd_25_Co_5_ alloys in 0.6 M NaCl. The polarization direction and positions of breakdown (E_bd_) and re-passivation potentials (E_rp_) are indicated by arrows. For the color interpretation of this figure, the reader is referred to the web version of this article.

A further insight into the peculiar corrosion behavior of the Al–Pd–Co alloys was obtained by potentiodynamic polarization. After the OCP measurement, a polarization scanning from −1000 mV to 0 mV (Ag/AgCl) was performed using a sweeping rate of 1 mV s^−1^. After reaching 0 mV (Ag/AgCl), the polarization direction was reversed and returned back to the initial potential (the direction of the polarization is indicated by open arrows in Figure 8). The resulting cyclic polarization curves are depicted in Figure 8. The forward curves are characterized by the corrosion minimum followed by an increase of the current density at potentials less negative than the corrosion potential. The current density increase was further followed either by stabilization (the Al_74_Pd_12_Co_14_ alloy) or even a slight decrease of the current density (the Al_70_Pd_25_Co_5_ alloy). This behavior indicates a passivation of the alloys. The transient behavior was further followed by a sudden current density increase at potentials less negative than −400 mV (Ag/AgCl), indicating a breakdown of the passive film. Upon reverse polarization, a re-passivation of the existing pits occurred. In order to compare equally polarized samples, we reversed the scanning at the fixed potential. The forward curves presented in Figure 8 have been analyzed by Tafel extrapolation [33]. The electrochemical parameters of the alloys (corrosion potential, corrosion current density, and breakdown potential) are listed in Table 3. A re-passivation potential obtained from the reverse curve is also presented. However, caution is required when comparing the individual re-passivation potentials of the alloys. The currents at the vertex were higher for the Al_74_Pd_12_Co_14_ alloy and this might have influenced the pit depth and local chemistry [33]. In order to obtain more comparable *E*_rp_ values, reversing the polarization at a constant current density would be necessary.

**Figure 9:**
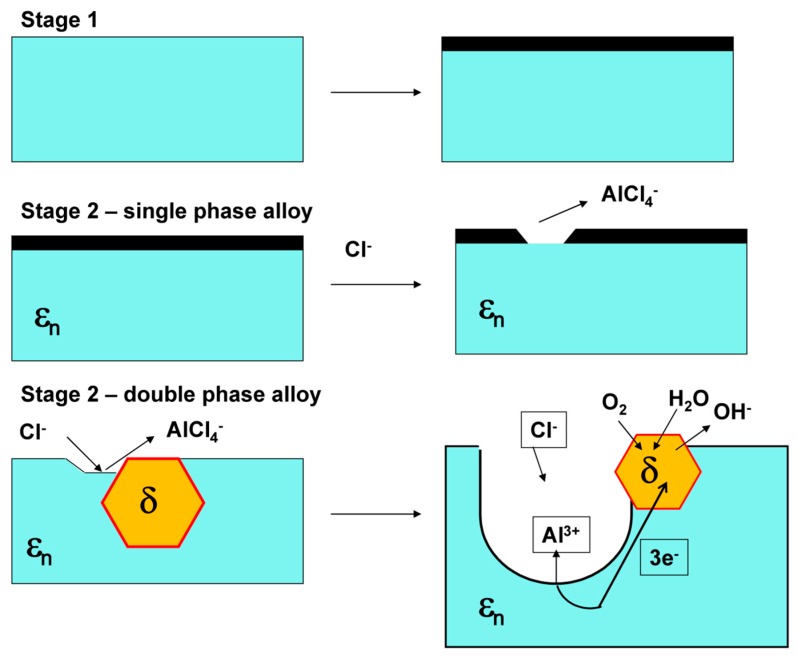
Pitting corrosion mechanism of the Al–Pd–Co alloys. For the color interpretation of this figure, the reader is referred to the web version of this article.

Based on the above-presented results, a corrosion mechanism of the Al–Pd–Co alloys has been postulated. The corrosion mechanism is depicted in Figure 9. Pitting is a highly localized form of corrosion that happens in the presence of halide anions, such as Cl^−^ [34]. Initially, a protective alumina scale has been formed on the sample surface, which is indicated by a current density plateau observed upon sample polarization for both alloys. This plateau is observed at potentials of −600 to −300 mV versus Ag/AgCl for Al_70_Pd_25_Co_5_ alloy, i.e., at potentials less negative than is the corrosion potential (Figure 8). In the presence of Cl^−^, however, this passive layer has been weakened. Aluminum forms unstable [AlCl_4_]^−^ complexes that dissolve in aqueous solutions. The dissolution of the protective alumina scale in NaCl leaves a naked alloy surface susceptible to further corrosion attack (Figure 9).

Interactions between co-existing phases in double-phase alloys may play an important role during corrosion [32,35,36]. Once the pitting potential is reached during sample polarization, the compact passivation layer becomes locally disrupted (Figure 9). As a result, Al^3+^ cations are released from the alloy into the solution in the course of the following reaction

Al → Al^3+^ + 3e^−^ (1)

Reaction (1) leads to positive charge enrichment within the dissolution zone [37]. As a consequence, Cl^−^ anions of the electrolyte rapidly migrate into the dissolution zone as presented in Figure 9. The released Al^3+^ cations become solvated by water molecules. Consequently, the hydrolysis of [Al(H_2_O)_4_]^3+^ in aqueous environment takes place in line with the following reaction [34]

(2) $$[\mathrm{Al}(H_{2}O{)}_{4}{]}^{3+}+H_{2}O\;↔\;[\mathrm{Al}(H_{2}O{)}_{3}(\mathrm{OH}){]}^{2+}+H_{3}O^{+}$$

Hydroxo complexes of Al may further react with chloride and water according to the following reactions

[Al(H_2_O)_3_(OH)]^2+^ + Cl^−^ → [Al(H_2_O)_2_(OH)Cl]^+^ + H_2_O (3)

[Al(H_2_O)_2_(OH)Cl]^+^ + H_2_O → [Al(H_2_O)(OH)_2_Cl] + H_3_O^+^ (4)

By these reactions, hydrogen cations are released into the pit. Their accumulation yields to a local pH decrease within the dissolution zone, which is known as a self-acidifying effect [34,37]. The presence of H^+^ in pits further accelerates the Al dissolution. At the cathode (δ, Figure 9), a reduction of water may take place in accordance with the following reaction

O_2_ + 2H_2_O + 4e^−^ → 4OH^−^ (5)

At the pit walls and possibly in their immediate vicinity, since pH is reduced due to hydrolysis reactions (2) and (4), the most likely prevailing cathodic reactions are

O_2_ + 4H^+^ + 4e^−^ → 2H_2_O (6)

or

2H^+^ + 2e^−^ → H_2_ (7)

The latter reaction takes place in the case of pH having fallen to very low values. As a result, emerging bubbles of H_2_ evolve on the alloy surface.

The results presented in Table 3 show that the OCP of the double-phase Al_70_Pd_25_Co_5_ alloy is located between the breakdown and re-passivation potentials. This is also manifested by the OCP oscillations observed between −330 mV (Ag/AgCl) and −400 mV (Ag/AgCl, Figure 7), indicating periodic breakdown and re-passivation events on the sample surface. These observations indicate that this alloy was in a localized corrosion stage already upon the sample’s immersion in the electrolyte, contrary to the single-phase Al_74_Pd_12_Co_14_ alloy. The passivation stage was found to be more pronounced in the case of the double-phase Al_70_Pd_25_Co_5_ alloy (Figure 8). This could be related to the presence of the noble δ phase in this alloy. For the mono-phasic Al_74_Pd_12_Co_14_ alloy, on the other hand, a higher corrosion current density (j_corr_) has been found, reflecting a higher dissolution rate. Moreover, a more negative corrosion potential for this alloy has been found, indicating a higher corrosion susceptibility. The above-reported differences in the corrosion behavior of the alloys could result from their different microstructures. More information about the specific corrosion attack of different SCIPs has been therefore obtained by investigating the alloys’ microstructures after electrode polarization.

**Figure 10:**
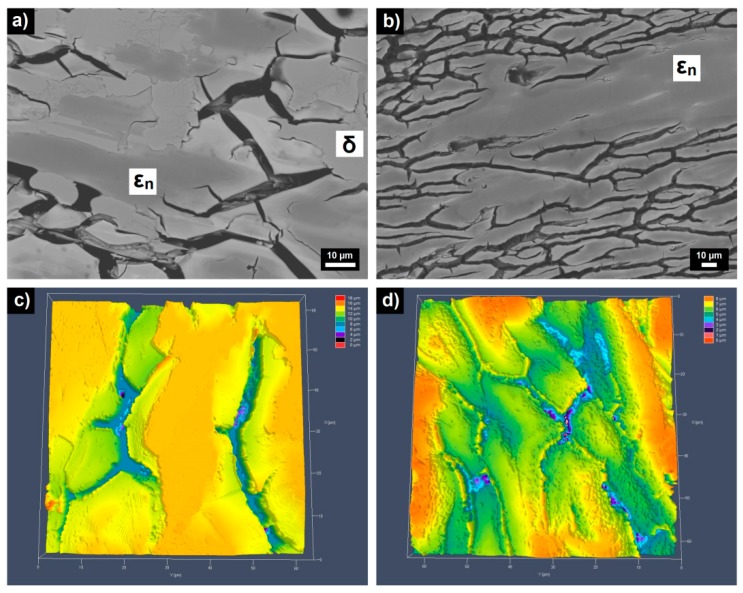
BEI/SEM images (**a**,**b**) and CLSM images (**c**,**d**) of as-polarized Al_70_Pd_25_Co_5_ (**a**,**c**) and Al_74_Pd_12_Co_14_ (**b**,**d**) alloys. Phases assigned to particular constituents are also marked. For the color interpretation of this figure, the reader is referred to the web version of this article.

The microstructures of the as-polarized alloys are documented in Figure 10. Metal concentrations of the phases after corrosion are summarized in Table 4. For both alloys, a preferential attack of ε_n_ was found. δ as a nobler phase in the Al_70_Pd_25_Co_5_ alloy has been retained. A de-alloying of Al from ε_n_ as well as formation of intermittent inter-penetrating channel networks have been observed in both alloys (Figure 10). In the single-phase Al_74_Pd_12_Co_14_ alloy, a higher density of intermittent inter-penetrating channels and pits has been found (Figure 10). Moreover, the channels formed a cross-linked network. This behavior is similar to the Al–Pd alloys, where the pits were observed in the interconnection between two channels [24]. In the Al_70_Pd_25_Co_5_ alloy, the pits were observed to be randomly distributed in the channels (Figure 10).

A dissolution of Al in the Al_70_Pd_25_Co_5_ alloy has been found (Table 4). Simultaneously, the Al concentration in ε_n_ decreased from 72.5 to 69.0 at.% (Table 4). In the Al_74_Pd_12_Co_14_ alloy, a decrease of Al concentration in ε_n_ from 73.9 to 71.1 at.% has been found. Thus, the level of Al de-alloying was higher in the double phase Al_70_Pd_25_Co_5_ alloy. Moreover, the pits found in this alloy were deeper compared to the Al_74_Pd_12_Co_14_ alloy. The formation of cracks observed in the as-polarized alloys could be governed by a combination of de-alloying kinetics and the release rate of internal stresses. As the electrochemical potential is raised in a positive direction, the dissolution rate of the alloy increases (Figure 8). This electrochemical force drives the surface at the de-alloying front further away from the equilibrium [37]. The removal of Al from the alloy phases leads to microcrack initiation. The residual stress accumulated in the alloys during rapid solidification is released during de-alloying.

**Figure 11:**
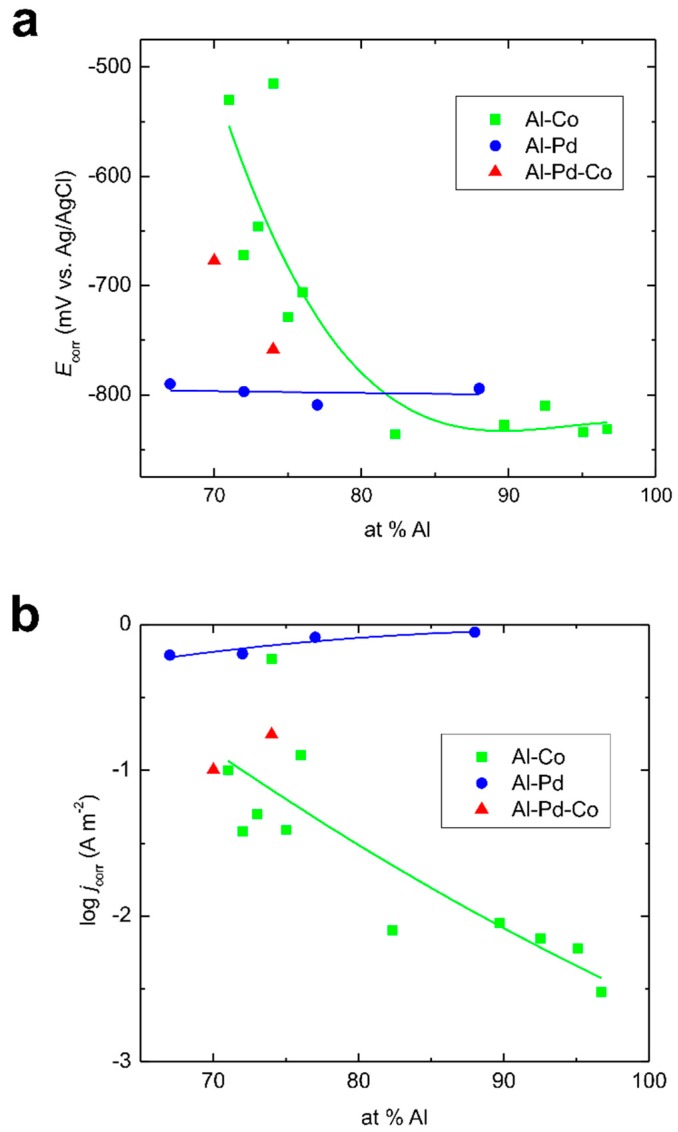
Corrosion potentials (**a**) and corrosion current densities (**b**) of as-solidified Al–Co, Al–Pd, and Al–Co–Pd alloys. Lines are a guide to the eyes only. For the color interpretation of this figure, the reader is referred to the web version of this article.

Corrosion potentials and corrosion current densities of the as-solidified Al–Co [17,18,20,21,38], Al–Pd [24], and Al–Pd–Co alloys are compared in Figure 11. The corrosion potentials of the Al–Co alloys show a significant dependence on the Al atomic fraction. They become more negative with increasing Al concentration. The corrosion potentials of the Al–Pd alloys, on the other hand, are relatively constant with respect to the alloy’s overall chemical composition. They are, in fact, more negative than the corrosion potentials of the remaining two groups of alloys. The corrosion currents of the Al–Pd alloys, on the other hand, are higher compared to the Al–Co and Al–Pd–Co alloys (Figure 11b). These observations suggest that the Al–Pd alloys are less corrosion-resistant compared to both the Al–Pd–Co and Al–Co alloys. The corrosion behavior of the Al–Pd–Co alloys is closer to the behavior of the Al–Co alloys. This observation is unexpected, since both alloy groups have a different chemical composition and phase constitution. Moreover, ε_n_, the preferentially corroding phase in the Al–Pd–Co alloys, is not present in the Al–Co alloys. Therefore, it can be suggested that Al_3_Co SCIPs are nobler compared to binary ε_n_ (Al_3_Pd). This is manifested by the less negative corrosion potentials of the Al–Co alloys compared to the Al–Pd alloys with a similar Al atomic fraction (Figure 11). Furthermore, the Co substitution for Pd significantly improves the corrosion resistance of ε_n_. As such, it is not the crystal structure of the phase, but its chemical composition, that plays a major role in the corrosion behavior.

**Figure 12:**
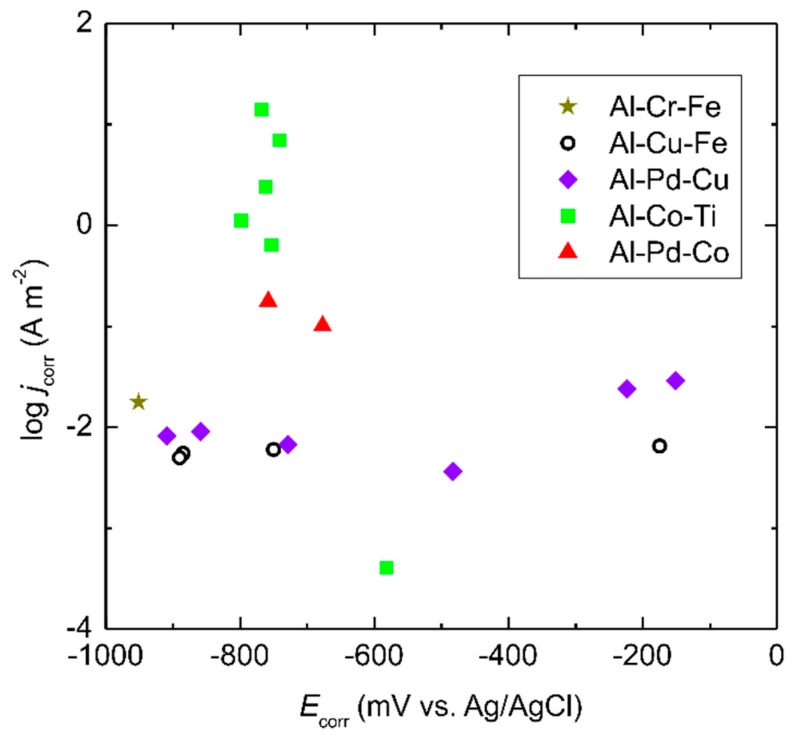
Corrosion current densities versus corrosion potentials of selected ternary Al-based complex metallic alloys (as-cast and as-annealed alloys only). For the color interpretation of this figure, the reader is referred to the web version of this article.

To further probe this hypothesis, we have plotted the corrosion data of other ternary Al–TM systems, found in the literature, together with those of the Al–Pd–Co system. The data survey [13,39,40,41,42] is presented in Figure 12. Although the data are scattered due to large variations in alloy chemical compositions, some trends can be identified. The as-solidified Al–Cu–Pd and Al–Cu–Fe alloys have lower corrosion currents compared to the Al–Pd–Co alloys [13,39]. The addition of Pd was found to slightly decrease the corrosion current of the Al–Cu alloys in chloride solution [39]. For the Al_4_Cu_9_ samples, however, not much effect from Pd has been seen [39]. The corrosion potentials of these alloys are found over a broad range of values. The scatter in E_corr_ values, however, could be caused by variations in their chemical composition. The Al–Cr–Fe alloy is also presented in Figure 12 [40]. This alloy has a more negative corrosion potential due to the absence of noble elements, such as Pd. Furthermore, it has a low corrosion current due to the presence of chromium, which forms a passive layer on the sample surface.

Interesting is the corrosion behavior of as-solidified Al–Co–Ti alloys [41]. These alloys have corrosion potentials comparable to those of the Al–Pd–Co alloys (Figure 12). The concentration of Ti in these alloys was fixed at 2 at.% and the atomic concentration of Co varied between 5 and 30 at.% (Al–xCo–2Ti alloys). As such, the materials design of these alloys had features typical of the Al–Co alloys [41,42]. In general, the corrosion currents of the Al–Co–Ti alloys are higher compared to those of the Al–Pd–Co alloys. An exception was found, however, for the Al–15Co–2Ti alloy since this alloy had a very low corrosion current. This difference is, however, attributable to the fact that the intermetallic particles present in this alloy (Al_9_Co_2_, Al_13_Co_4_, and Al_3_Ti) were of different morphologies and volume fractions compared to the remainder of the alloys [41]. These observations indicate that the specific Co concentrations may greatly improve the corrosion performance of the Al–TM alloys. The ε_n_ phase in the Al–Pd–Co alloys contains a significant amount of Co. The Co additions thus contribute to the corrosion resistance of the Al–Pd–Co alloys and this is especially obvious in the case of the double phase Al_70_Pd_25_Co_5_ alloy.

Al_3_Ti and Al_3_Fe are noble intermetallic phases with respect to the aluminum matrix [36]. The results presented in this work show that Al_3_Co is also relatively noble. These phases are nobler compared to binary ε_n_ (Al_3_Pd). Co substitution for Pd thus significantly improves the corrosion resistance of ε_n_. As such, it is not the crystal structure of the phase, but its chemical composition, that plays a major role in the corrosion behavior. The electrochemical behavior of constituent phases may change over time. In a recent study, Zhu et al. studied the evolution of corrosion behavior of intermetallic phases in Al alloys over time [43]. At the early stages, the corrosion attack occurred in the form of de-alloying. However, as the time progressed, the particles became nobler as a result of Al dissolution [43]. This particle ennoblement may accelerate the galvanic dissolution of the surrounding matrix. The corrosion behavior of constituent phases may also change as a result of long-term annealing. The long-term annealing causes element redistribution and reduces stresses accumulated during rapid solidification [19]. A comparative study of as-annealed, near-equilibrium Al–Pd–Co alloys is planned and results will be reported in a future publication.

## 4. Conclusions

In this work, the corrosion performance of as-solidified Al_70_Pd_25_Co_5_ and Al_74_Pd_12_Co_14_ alloys was studied by open circuit potential measurements and potentiodynamic polarization in aqueous NaCl (3.5 wt.%), following an in-depth structural characterization of the alloys. The alloys were prepared by arc-melting of Pd, Al, and Co lumps in argon. Based on the results, the following conclusions can be presented:

1. The Al_74_Pd_12_Co_14_ alloy was a single-phase alloy composed of ε_n_. In this alloy, a combination of three ε_n_ structures was identified: ε_6_, ε_16_, and ε_28_.
2. The Al_70_Pd_25_Co_5_ alloy was a double-phase alloy composed of ε_n_ and δ (Al_3_Pd_2_). In this alloy, two ε_n_ structures were identified: ε_6_ + ε_28_.
3. Marked open circuit potential oscillations of the Al_70_Pd_25_Co_5_ alloy have been observed, indicating individual breakdown and re-passivation events on the sample surface. A preferential corrosion attack of ε_n_ was found. Binary δ phase (Al_3_Pd_2_) was less affected by corrosion.
4. De-alloying of Al from ε_n_ and formation of intermittent inter-penetrating channel networks occurred in both alloys.
5. The corrosion attack of the Al_74_Pd_12_Co_14_ alloy was more significant compared to the Al_70_Pd_25_Co_5_ alloy and resulted in the formation of a de-alloyed and highly porous metallic network. The corrosion susceptibility of ε_n_ could be further utilized in preparing porous Pd–Co alloys with possible catalytic activity.
6. The Co substitution for Pd significantly improves the corrosion resistance of ε_n_. As such, it is probably not the crystal structure of the phase, but its chemical composition, that plays a major role in the corrosion behavior.
7. Specific Co concentrations may greatly improve the corrosion performance of the Al–TM alloys.

## Figures and Tables

**Table 1:** Overview of selected binary and ternary phases occurring in the Al–Pd–Co system and related binaries [3,8].

Phase	Space Group/Symmetry	Lattice Parameter			
		a (nm)	b (nm)	c (nm)	β (°)
Al–Pd–Co system					
W	*Pmn*2_1_	2.36	0.82	2.07	-
Y_2_	*Immm*	1.5451	1.2105	0.7590	-
U	*C*121, *C*1*m*1 or *C*12/*m*1	1.9024	2.9000	1.3140	117.26
V	*P*121, *P*1*m*1 or *P*12/*m*1	1.0068	0.3755	0.6512	102.38
F	$P2_{1}/a\bar{3}$	2.4397	-	-	-
C_2_	$Fm\bar{3}$	1.5507	-	-	-
ε_16_	*Amm*2	2.35	1.68	3.26	-
ε_22_	orthorhombic	2.35	1.68	4.49	-
ε_34_	orthorhombic	2.35	1.68	7.01	-
Al–Pd system					
ε_6_	*Pna*2_1_	2.35	1.68	1.23	-
ε_28_	*C*2*mm*	2.35	1.68	5.70	-
Al_3_Pd_2_ (δ)	$P\bar{3}m1$	0.4227	-	0.5167	-
AlPd (β)	$Pm\bar{3}m$	0.3036	-	-	-
Al–Co system					
Al_9_Co_2_	*P*2_1_/*a*	0.85565	0.6290	0.62130	94.76
O-Al_13_Co_4_	*Pmn*2_1_ or *Pnmn*	0.8158	1.2347	1.4452	-
M-Al_13_Co_4_	*C*2/*m*	1.5173	0.81090	1.2349	107.84
Z-Al_3_Co	*monoclinic*	3.984	0.8148	3.223	107.97
Al_5_Co_2_	*P*6_3_/*mmc*	0.76717	-	0.76052	-
AlCo (β)	$Pm\bar{3}m$	0.2854	-	-	-

**Table 2:** Metal concentrations and phase assignments of microstructure constituents observed in as-solidified Al_70_Pd_25_Co_5_ and Al_74_Pd_12_Co_14_ alloys.

Alloy	Alloy Condition	Identified Phase/Structure	Element Concentration (at.%)			Volume Fraction (%)
			Al	Pd	Co	
Al_70_Pd_25_Co_5_	as-solidified	ε_n_/ε_6_ + ε_28_	72.5 ± 0.1	18.9 ± 0.3	8.6 ± 0.3	77
		δ	59.8 ± 0.2	39.7 ± 0.4	0.5 ± 0.2	23
Al_74_Pd_12_Co_14_	as-solidified	ε_n_/ε_6_ + ε_16_ + ε_28_	73.9 ± 1.0	12.0 ± 5.5	14.1 ± 4.6	100

**Table 3:** Electrochemical parameters of as-solidified Al_70_Pd_25_Co_5_ and Al_74_Pd_12_Co_14_ alloys. Corrosion potentials (E_corr_) and corrosion current densities (j_corr_) were obtained by Tafel extrapolation of forward curves (Figure 8).

Alloy	OCP (mV vs. Ag/AgCl)	E_corr_ (mV vs. Ag/AgCl)	j_corr_ (A m^−2^)	E_bd_ (mV vs. Ag/AgCl)	E_rp_ (mV vs. Ag/AgCl)
Al_70_Pd_25_Co_5_	−370 ± 35	−677	0.101	−332	−447
Al_74_Pd_12_Co_14_	−607 ± 9	−758	0.176	−393	−540

**Table 4:** Metal concentrations and phase assignments of the as-polarized Al–Pd–Co alloys. Differences in metal concentrations between as-polarized and as-solidified alloys are also presented (compare with data in Table 2).

Alloy	Identified Phase/Structure	Element concentration (at.%)					
		Al	Δ(Al)	Pd	Δ(Pd)	Co	Δ(Co)
Al_70_Pd_25_Co_5_	ε_n_/ε_6_ + ε_28_	69.0 ± 0.3	−3.5	22.2 ± 0.4	+ 3.3	8.8 ± 0.4	-
	δ	60.0 ± 0.3	-	39.4 ± 0.3	-	0.6 ± 0.2	-
Al_74_Pd_12_Co_14_	ε_n_/ε_6_ + ε_16_ + ε_28_	71.1 ± 0.9	−2.8	14.3 ± 5.3	+ 2.3	14.6 ± 4.3	-
